# A Useful Disinfectant

**Published:** 1898-01

**Authors:** 


ARTICLE IX.

### A USEFUL DISINFECTANT.

In its issue of December 11, the Maryland Medical Journal gives some interesting details as to the use of formaldehyde gas, the disinfectant which has recently come into favor. The disinfection of rooms and clothing by means of the fumes of sulphur and by solutions of carbolic acid and chloride of lime is generally very difficult and often ineffective. The sulphur fumes lack power of penetration, being inoperative unless the air or clothing to be disinfected is damp. The other disinfectants named are effective only when the infected object is soaked in their solutions. But for formaldehyde gas it is claimed that it will not only destroy any disease germs left in the air of a room by a recent patient, but will also destroy any germs which may have remained in the folds of draperies or hangings, and even cloths hung up in the room can probably be rendered free from infectious bacteria. The gas has the advantage over other substances, it is said, that it does not alter or injure the colors of fabrics, furniture or other articles of household use. The disinfectant is commonly sold under the name "formalin," which is a 40 per cent solution of formaldehyde gas. The formalin is heated in a vessel with chloride of colcium, with the result of disengaging the gas in a dry state. By means of a metallic tube the gas is then introduced into the infected room through the keyhole. By way of preparation all openings should be stopped so as to confine the gas closely within the space to be disinfected. A quart of the formalin should be evaporated for each 1,000 cubic feet of space. The gas is not poisonous, but irritates the eyes and throat. A room fumigated with it should remain closed for twenty-four hours. "Germs of all the various diseases," says the Medical Journal, "such as cholera, typhoid fever, bubonic plague, anthrax, tuberculosus, diphtheria and the germs of ordinary inflammation have been exposed in closed rooms to the action of this gas. Pieces of cloth soaked in cultures of disease bacteria have been exposed freely in the room, or covered by blankets and carpets. Germs have been placed between the leaves of books, put into envelops in the pockets of clothing, or even placed in the interior of mattresses. It has been found that after exposure to this gas, not only were the germs on the surface destroyed, but if enough gas was present the germs covered by blankets or even in the depths of a mattress, were killed." The gas is already being widely used for routine disinfection.

